# Trans-Kingdom Conjugation within Solid Media from *Escherichia coli* to *Saccharomyces cerevisiae*

**DOI:** 10.3390/ijms20205212

**Published:** 2019-10-21

**Authors:** Maximillian P. M. Soltysiak, Rebecca S. Meaney, Samir Hamadache, Preetam Janakirama, David R. Edgell, Bogumil J. Karas

**Affiliations:** 1 Department of Biology, The University of Western Ontario, London, ON N6A 5B7, Canada; msoltys4@uwo.ca (M.P.M.S.); shamadac@uwo.ca (S.H.); 2 Designer Microbes Inc., London, ON N5Z 3N2, Canada; rmeaney2@uwo.ca (R.S.M.); preetam.janakirama@gmail.com (P.J.); 3 Department of Biochemistry, Schulich School of Medicine and Dentistry, The University of Western Ontario, London, ON N6A 5C1, Canada; dedgell@uwo.ca

**Keywords:** conjugation, solid media, *Saccharomyces cerevisiae*, trans-kingdom, *Escherichia coli*, pTA-Mob, yeast assembly

## Abstract

Conjugation is a bacterial mechanism for DNA transfer from a donor cell to a wide range of recipients, including both prokaryotic and eukaryotic cells. In contrast to conventional DNA delivery techniques, such as electroporation and chemical transformation, conjugation eliminates the need for DNA extraction, thereby preventing DNA damage during isolation. While most established conjugation protocols allow for DNA transfer in liquid media or on a solid surface, we developed a procedure for conjugation within solid media. Such a protocol may expand conjugation as a tool for DNA transfer to species that require semi-solid or solid media for growth. Conjugation within solid media could also provide a more stable microenvironment in which the conjugative pilus can establish and maintain contact with recipient cells for the successful delivery of plasmid DNA. Furthermore, transfer in solid media may enhance the ability to transfer plasmids and chromosomes greater than 100 kbp. Using our optimized method, plasmids of varying sizes were tested for transfer from *Escherichia coli* to *Saccharomyces cerevisiae*. We demonstrated that there was no significant change in conjugation frequency when plasmid size increased from 56.5 to 138.6 kbp in length. Finally, we established an efficient PCR-based synthesis protocol to generate custom conjugative plasmids.

## 1. Introduction

Conjugation is a widespread bacterial mechanism for DNA transfer and a major contributor to the spread of antibiotic resistance and virulence factors [1]. Through the advent of recombinant DNA technology, conjugation has been adapted for extensive use in biotechnology as a simple alternative for DNA transfer to a broad range of recipient species. Although initially described as a prokaryotic phenomenon, conjugal transfer is not limited strictly to bacterial recipients. Trans-kingdom conjugation is observed in nature in the form of T-DNA transfer from *Agrobacterium* species to plants [2,3]. For bioengineering purposes, various bacterial donor species have been used to deliver DNA to eukaryotic recipients such as the yeast *Saccharomyces cerevisiae* [4,5,6,7,8,9], algal diatoms *Phaeodactylum tricornutum* and *Thalassiosira pseudonana* [9,10,11,12,13], and mammalian cells [14,15,16,17].

Conventional transformation techniques, such as electroporation and chemical transformation [18], have been developed for many species, yet suffer from some drawbacks [19]. For one, these methods require pure, intact DNA molecules, which can be challenging to isolate, especially large molecules (>100 kbp) which are prone to damage from shear forces during purification and handling [20]. Other techniques, such as viral transformation or cell fusion, can be used to transfer DNA directly to destination cells. Nonetheless, both techniques are limited to specific donor-recipient organisms, and in the case of viral transformation, by the size of the DNA that can be transferred. Conjugation provides an alternative approach to achieve delivery of either self-transferring (*cis*) or mobilizable (*trans*) plasmids into a wide range of recipient species. For *in situ* applications, conjugation is especially useful when delivering DNA to soil rhizospheres [21,22] or gut microbiomes [23,24]. Although easy to use when transferring DNA between prokaryotic cells, optimal conditions for conjugal transfer to eukaryotes remain poorly explored. Furthermore, while it has been previously demonstrated that plasmids up to 875 kbp in size can be transferred to prokaryotic recipients, the upper size limit of conjugal transfer to eukaryotes has yet to be determined [25].

The majority of conjugation systems use a subfamily of the type IV secretion system (T4SS) to export DNA to recipient cells, however, the composition and structure of T4SS complexes differ among the identified conjugative plasmid groups [26,27]. Some conjugation systems, such as IncF, IncH, and IncI plasmids, transfer DNA efficiently in liquid media, while others, including the IncN, IncM, IncP, and IncW plasmids, achieve higher DNA transfer frequencies on solid media [28]. It is suspected that the ability to transfer DNA in different environmental conditions is related to variation in pilus formation, structure, and stability of cells during the conjugation process. If conjugation would occur within solid media, as opposed to on the surface or in liquid, the environment may be more stable. The increased stability could prolong the time during which a donor bacterium is attached to the recipient cell, and therefore may be more conducive to the transfer of large plasmids or even whole chromosomes. A protocol where the conjugal transfer occurs within solid media may also expand the use of conjugation as a tool for DNA transfer to species that require semi-solid or solid media for growth [29,30,31]. Furthermore, such a protocol may permit more accurate enumeration of transconjugants by avoiding the use of a spreader while plating cells on selective media [32].

We developed and optimized a simple protocol for *cis* and *trans* conjugal transfer from *E. coli* to *S. cerevisiae* within solid media. Both species are established laboratory model organisms for cloning and storage of DNA and are widely studied due to their influence on human health [33,34]. Using the newly developed solid media conjugation protocol, we transferred plasmids to *S. cerevisiae* ranging in size from 18.1 to 138.6 kbp. Notably, we showed that there was no significant change in conjugation frequency as the size of the plasmids increased from 56.5 to 138.6 kbp in length. We also established an efficient and reproducible PCR-based synthesis pipeline to generate the conjugative plasmid pTA-Mob 2.0, a derivative of the RK2-based IncP plasmid pTA-Mob [35]. Both tools improve how we build and deliver DNA via conjugation from prokaryotic to eukaryotic cells.

## 2. Results

### 2.1. PCR-Based Synthesis of Conjugative Plasmid pTA-Mob 2.0

**Figure 1:**
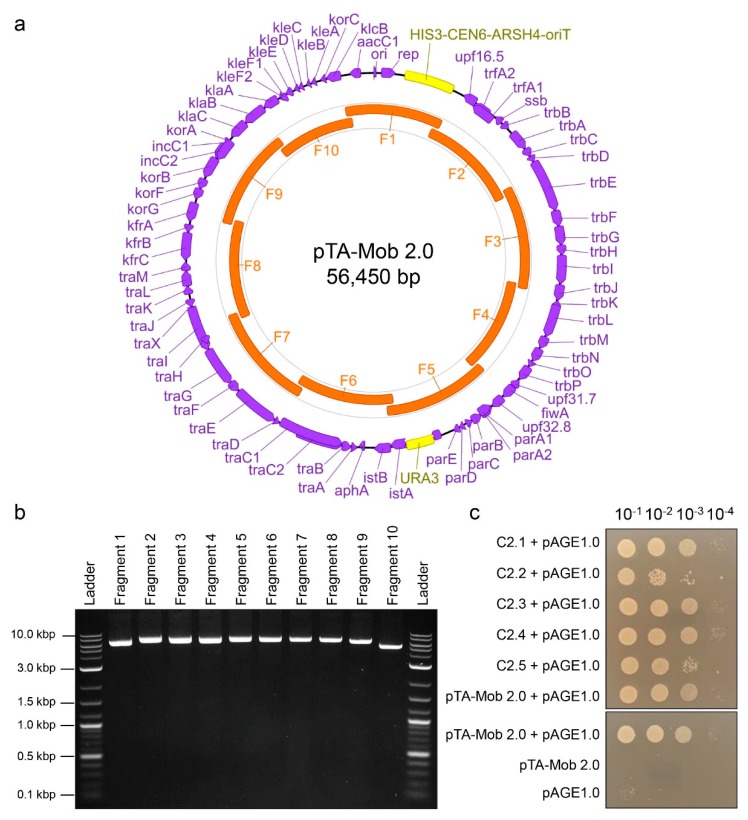
Optimized PCR-based synthesis of plasmid pTA-Mob 2.0: (**a**) Plasmid map of pTA-Mob 2.0 with coding regions (CDS) annotated in purple, and inserted regions (yeast elements and origin of transfer as described in this paper) in yellow. Regions highlighted in orange indicate the 10 overlapping fragments used in optimized assembly. CDS annotations were transferred from the Birmingham IncP-alpha isolate RK2 [36] and then individually confirmed via BLAST against the NCBI non-redundant protein sequences database (BLASTx). The figure was generated using Geneious version 2019.2, created by Biomatters. (**b**) Agarose gel electrophoresis of pTA-Mob 2.0 amplified as ten overlapping fragments and (**c**) partial conjugation results of newly assembled pTA-Mob 2.0 plasmids (colonies C2.1 to C2.5) to recipient *E. coli* containing the pAGE1.0 plasmid. As a positive control, we used parental pTA-Mob 2.0 conjugating to pAGE1.0, and as negative controls we used donor pTA-Mob 2.0 or recipient pAGE1.0 alone.

In previous studies, the non-mobilizable helper plasmid pTA-Mob was used to transfer destination plasmids from bacteria to recipient bacteria [35], algae [10,12,13], and yeast [9]. The pTA-Mob plasmid encodes the machinery required for conjugal transfer of plasmids that contain an origin of transfer (oriT) [35]. For this study and future applications, we designed a method to build an alternative version of pTA-Mob, named pTA-Mob 2.0, that can self-mobilize (*cis*) and replicate in both *E. coli* and *S. cerevisiae*. To this end, we used PCR to initially amplify pTA-Mob as nine overlapping fragments along with three additional fragments: a *S. cerevisiae URA3* gene; a gene cassette containing the *S. cerevisiae HIS3* gene, a yeast centromere (*CEN6*), and an autonomously replicating sequence (*ARSH4*); and an oriT cassette (Supplementary Figure S1). After assembling the fragments into whole plasmids in yeast, we transferred the DNA to *E. coli* and tested individual colonies for conjugation on top of agar to recipient *E. coli*. For subsequent work, we selected one colony harboring pTA-Mob 2.0 and isolated the plasmid for optimization of a simplified PCR-based plasmid synthesis pipeline. The reason for this optimization step was to simplify the assembly from twelve to ten fragments as well as to obtain cleaner PCR products. This improved protocol functions to accelerate the creation of designer pTA-Mob 2.0 variants. We designed new primers to amplify the plasmid as ten approximately equal-sized overlapping fragments (Figure 1a,b). To eliminate template DNA from the PCR amplified fragments, the inclusion of which would result in false positive assemblies, all reactions were treated with DpnI restriction enzyme. Once again, following yeast assembly, we transferred DNA to *E. coli* and tested for conjugation to a recipient *E. coli* strain. This initial *E. coli* to *E. coli* conjugation analysis provided a rapid approach to evaluate successfully assembled plasmids before testing conjugal transfer to eukaryotic cells. Of the colonies tested, 23 out of the 30 donor colonies conjugated at least as well as the parental pTA-Mob 2.0 (Figure 1c, Supplementary Figure S2). We sequenced plasmids isolated from three out of the 23 colonies that conjugated at least as well as the parental pTA-Mob 2.0 and found three, four, and twelve mutations in each respective colony (Supplementary Table S2).

### 2.2. Development and Optimization of Conjugation within Solid Media

The initial protocol for conjugation in solid media was inspired by the method for direct transfer of DNA from bacteria to yeast, where a polyethylene glycol-treated mixture of bacteria and yeast spheroplasts is suspended in molten agar media and then plated in a Petri dish [20,37]. For the development of the conjugation protocol, we used intact yeast cells and did not treat the mixture with polyethylene glycol. Early attempts performing the conjugation protocol resulted in inconsistent results (data not shown) prompting us to develop an optimized protocol. Optimization parameters included: (i) molten agar media temperature, (ii) molten agar media agar concentration, and (iii) volumes of *E. coli* and *S. cerevisiae* cell suspensions harvested at various optical densities (ODs). We used the optimal value for each parameter in subsequent optimization experiments.

**Figure 2:**
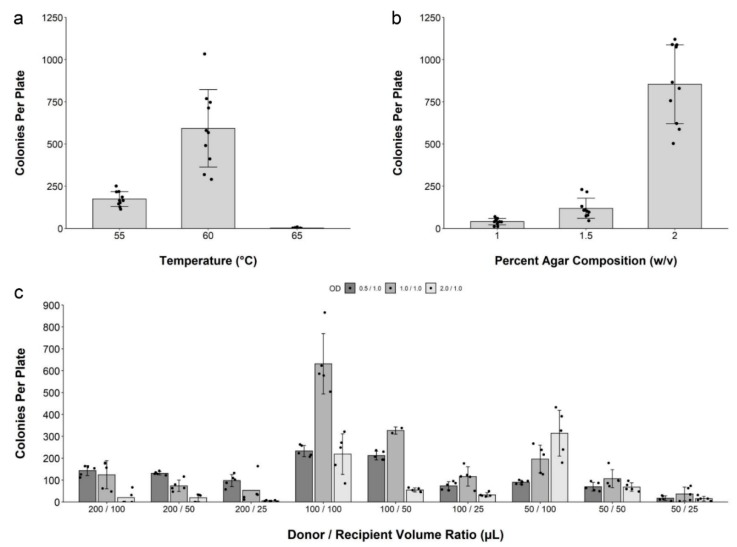
Optimization of the conjugation protocol in solid media. Transconjugant yeast colonies per plate yielded from optimization experiments testing: (**a**) molten agar media temperature, (**b**) Molten agar media agar composition prior to the addition of cell mixture, and (**c**) volumes of *E. coli* and *S. cerevisiae* cell suspensions harvested at various optical densities (OD_600_).

First, when testing the effect of molten agar media temperature, we found that a temperature of 60 °C was optimal for transconjugant colony formation (Figure 2a). For the initial agar concentration, 2% agar (*w*/*v*) was the most conducive to colony formation (Figure 2b). Higher agar concentrations were not practical due to the rapid solidification of the media. We then tested 27 combinations of various volumes of donor and recipient cells harvested at different ODs and counted the number of transconjugant yeast colonies per plate (Figure 2c). On the basis of this experiment, mixing 100 µL of donor and recipient cell suspensions, harvested at an OD_600_ of 1.0, resulted in the highest number of transconjugant colonies.

**Figure 3:**
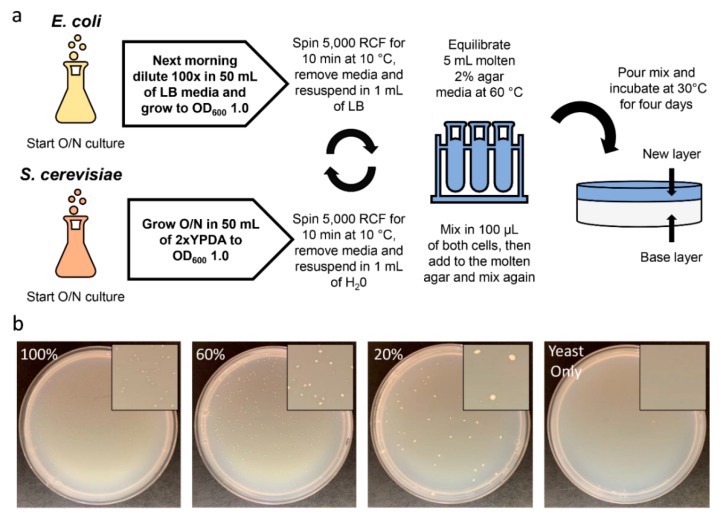
Optimized protocol for conjugation in solid media: (**a**) Schematic for the final protocol for conjugation from donor *E. coli* to recipient *S. cerevisiae* within solid media and (**b**) comparison of using 100%, 60%, and 20% of the optimized 100 µL/100 µL donor and recipient cell ratio. Yeast only setup was used as a negative control.

We summarized the optimization results in a final protocol for the conjugation in solid media method, as illustrated in Figure 3a and Supplementary Note S1. Using this optimized protocol, transconjugant colony formation is highest in number, however, the size of the colonies was small. Using smaller volumes of the final cell mix (100 µL/100 µL donor and recipient cells), such as 60% or 20%, leads to the formation of fewer but larger colonies (Figure 3b).

### 2.3. Conjugation of IncP-Based Plasmids of Increasing Size

**Figure 4:**
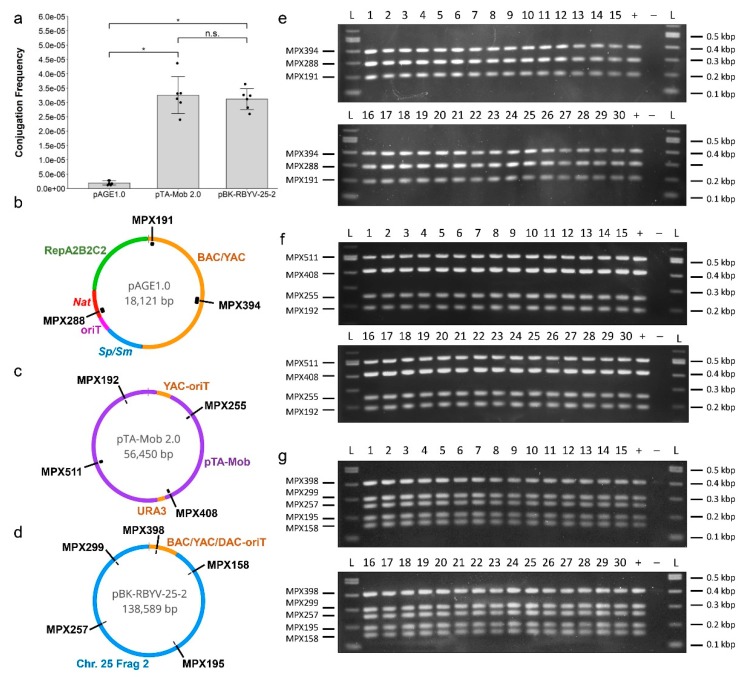
Conjugal transfer of plasmids increasing in size. (**a**) Conjugation frequency from donor *E. coli* containing either pTA-Mob 2.0 alone or pTA-Mob with either pAGE1.0 or pBK-RBYV-25-2 to recipient *S. cerevisiae.* *: indicates significant difference in conjugation frequency as determined by pairwise T tests at a significance level of 0.05 with Holm correction and n.s.: no significant difference. The *p*-values for each comparison are as follows: pAGE1.0–pTA-Mob 2.0, *p* = 1.4 × 10^−4^; pAGE1.0–pBK-RBYV-25-2, *p* = 1.1 × 10^−5^; pTA-Mob 2.0–pBK-RBYV-25-2, *p* = 0.65. (**b**–**d**) Plasmid maps and Multiplex PCR primer locations of (**b**) pAGE1.0 (BAC/YAC: bacterial/yeast artificial chromosome; Sp/Sm: streptomycin-spectinomycin resistance marker for selection in *S. meliloti*; oriT: origin of transfer; *Nat*: N-acetyl transferase gene providing resistance to nourseothricin; and repA2B2C2: origin of replication for *Sinorhizobium meliloti*), (**c**) pTA-Mob 2.0 (YAC-oriT: yeast artificial chromosome and origin of transfer; pTA-Mob: plasmid backbone with all genes from the pTA-Mob plasmid; *URA3*: auxotrophic marker for selection in yeast), and (**d**) pBK-RBYV-25-2 (BAC/YAC/DAC-oriT: bacterial/yeast/diatom artificial chromosome and origin of transfer, Chr. 25 Frag 2: partial *P. tricornutum* chromosome 25 with additional *ARSH4* sequence and kanamycin selection added to it as described in [38]). (**e**–**g**) Diagnostic Multiplex PCR for 30 transconjugant *S. cerevisiae* colonies containing: (**e**) pAGE1.0, (**f**) pTA-Mob 2.0, or (**g**) pBK-RBYV-25-2. For (**e**–**g**), the expected size for each multiplex fragment is indicated in the label (for example, the expected size for MPX288 amplification from pAGE1.0 is 288 bp); “+” indicates the positive control, where appropriate isolated original plasmid DNA was used, and “−” indicates the negative control, where no template DNA was added to the PCR reaction. Note, a faint band can be seen around 400 bp in the negative control of the Multiplex PCR for pBK-RBYV-25-2, which is most likely nonspecific amplification.

Using the optimized protocol, we tested the following conjugation of plasmids of increasing size: pAGE1.0 (18.1 kbp) [9], pTA-Mob 2.0 (56.5 kbp), and pBK-RBYV-25-2 (138.6 kbp). The pBK-RBYV-25-2 plasmid was previously published [38] where the pBK-RBYV backbone was used to clone part of the *P. tricornutum* chromosome 25. To transfer pAGE1.0 and pBK-RBYV-25-2, the donor *E. coli* also contained the pTA-Mob helper plasmid to allow for mobilization of the plasmids in *trans* [35]. Surprisingly, conjugation with the larger plasmids produced more yeast colonies than with the smaller pAGE1.0 plasmid. Conjugation frequencies were then calculated using the number of transconjugant colonies divided by the colony-forming units obtained from the recipient *S. cerevisiae* serial dilutions. The conjugation frequencies for pAGE1.0, pTA-Mob 2.0, and pBK-RBYV-25-2 to *S. cerevisiae* were determined to be 1.9 × 10^−6^, 3.3 × 10^−5^, and 3.1 × 10^−5^, respectively (Figure 4a).

To verify that entire plasmids were successfully transferred to yeast, we consecutively restreaked the yeast colonies three times every two days on selective media containing ampicillin to remove any leftover donor *E. coli*. After the second passage, a portion of each colony was streaked onto an LB plate containing gentamicin and incubated at 37 °C overnight. The LB plate did not yield any *E. coli* colonies after incubation, confirming that any leftover donor *E. coli* was eliminated during the passage of the yeast colonies. Plasmid DNA from the yeast transconjugants was then isolated after the third passage by lysing the cells in TE buffer and used for diagnostic Multiplex PCR (Figure 4e–g). For each plasmid, we tested 30 individual recipient colonies, and all 90 colonies yielded bands of the expected sizes as shown in Figure 4e–g. The multiplex PCR results indicated that complete conjugal transfer of each plasmid was achieved for every colony tested and suggested that the frequency of conjugation to *S. cerevisiae*, when using either the pTA-Mob (*trans*) or pTA-Mob 2.0 (*cis*) system, does not decrease as plasmid size increases within the tested range using the solid media conjugation protocol.

## 3. Discussion

The development of engineered conjugative plasmids such as pTA-Mob [35], pLS20 [39], pRK2013 [19], pRH210 [40], and RP4 [41] provides a simple alternative for DNA transfer to prokaryotic and eukaryotic recipient cells. As recently demonstrated, the development of conjugation methods for species such as the eukaryotic algae *P. tricornutum* and others allows for rapid advances in genetic engineering of these organisms [10,12,13]. In this study, we aimed to develop a pipeline for building conjugative plasmids and subsequently demonstrate that such plasmids can be efficiently delivered from a bacterial donor to a eukaryotic recipient within solid media. To achieve this, we used *E. coli* as a conjugative donor and the model yeast *S. cerevisiae* as the eukaryotic recipient. Trans-kingdom conjugation to *S. cerevisiae* from *E. coli* had already been demonstrated in liquid conditions and on the surface of solid media, providing a basis for the creation of the conjugation-in-solid-media protocol [8,9].

First, we developed a PCR-based plasmid synthesis pipeline for building conjugative plasmids that can propagate in *E. coli* and yeast. Using this method, we found that 23 out of 30 plasmid clones tested were able to conjugate at least as well as the parental pTA-Mob 2.0 clone. After sequencing three of these plasmids, an average of 6.33 mutations per 56.5 kbp plasmid was found. It has been previously demonstrated that Takara PrimeSTAR GXL polymerase has an error rate of about 8.4 × 10^−6^ substitutions per base per PCR doubling [42]. If we consider each PCR cycle in the optimized protocol as a doubling, the error rate found in our plasmids was determined to be similar to the literature value, at around 4.5 × 10^−6^ mutations per base per PCR doubling, corresponding to a mutation about every 8.9 kbp. Aside from the PCR step, some of these mutations may have arisen during plasmid assembly in yeast and or during propagation of plasmids in yeast and or *E. coli*. If desired, mutations could be eliminated by first cloning each correct fragment flanked by preferred unique restriction sites in the plasmid of interest. Fragments could then be easily released by restriction digest, followed by yeast assembly. Since this pipeline utilizes PCR fragments for assembly, incorporating modular components and customizing plasmid based on pTA-Mob 2.0 is rapid and straightforward. Additional components can be incorporated into the assembly mixture if there is built-in homology on the terminal ends of the fragment. Homology can be easily introduced during amplification by designing primers that contain a “hook” with sequence complementarity to the flanking DNA fragments. To delete components, such as an undesirable gene, a single fragment can be amplified as two separate fragments flanking the unwanted DNA region, creating a seamless deletion of the target. Not only can the pipeline generate functional conjugative plasmids, as indicated with the creation and testing of pTA-Mob 2.0, it can be adapted for use with any designer customizable plasmid.

During the optimization process, we found that the ideal holding temperature for the molten agar media, until the cell mixture was added, was 60 °C. Previous studies using different bacterial species have also demonstrated that a “heat shock” step during conjugation results in improved conjugation efficiencies [43,44,45], however, it remains unclear as to why the heat shock at 60 °C yields an increased number of transconjugant *S. cerevisiae* colonies. Next, we demonstrated that the number of transconjugant colonies per plate increased when a higher agar composition was used, producing the best results with 2% agar. As agar concentration increases, the movement of cells is restricted, and therefore may provide additional stability of the pili during the formation of cell-to-cell attachments between bacteria and yeast.

A molten media composition of higher than 2% agar was difficult to pour and impacted the ability to ensure the top layer was even for the conjugation plates. If needed, higher percentage compositions of the media could be achieved using low-melting-point agarose, to avoid premature solidification of the top layer.

Next, we demonstrated that cell density had a substantial effect on yeast colony formation. After optimization, the most consistent conditions for transconjugant colony yields were achieved when both donor and recipient cultures were harvested at an OD_600_ of 1.0, and 100 µL of each cell resuspension was used. For experiments where faster growth of yeast colonies is beneficial or to obtain larger transconjugant yeast colonies, decreasing the volume of cell resuspensions used can yield such a result. During the early optimization process, it was found that the thickness in the top agar layer had an impact on the formation of transconjugant yeast colonies (data not shown). Thus, the total top agar plus cell mixture volume must be 6 mL.

Once the protocol for conjugation within solid media was optimized, three plasmids of increasing size were then tested, pAGE1.0 (18.1 kbp), pTA-Mob 2.0 (56.5 kbp), and pBK-RBYV-25-2 (138.6 kbp). For donor *E. coli* containing pAGE1.0 and pBK-RBYV-25-2, the plasmids were mobilized using pTA-Mob as a helper plasmid for *trans* conjugation. In comparison, pTA-Mob 2.0 can self-transfer via *cis* conjugation. As both types of plasmids are no longer mobilizable once in *S. cerevisiae*, the difference in colony yields would not be confounded by the re-conjugation of recipients in the *cis* setup. It has been previously stated that conjugation efficiency decreases as plasmid size increases [5,46]. In this study, however, there was found to be no significant change when comparing the transconjugant yields between the 56.5 kbp plasmid (pTA-Mob 2.0) and the 138.6 kbp plasmid (pBK-RBYV-25-2). From the multiplex PCR reactions, we were able to confirm that the complete transfer of all three plasmids occurred in every colony screened. With no significant change in conjugation frequency between 56.5 to 138.6 kbp plasmids, and complete transfer of each plasmid confirmed, the upper size limit of conjugal transfer to eukaryotic cells has not yet been met. As for why pAGE1.0, a relatively small plasmid, did not conjugate as well as the larger plasmids, additional investigation is required.

Prior to this study, conjugation within solid media to yeast had not been demonstrated. This method allows for the transfer plasmids of at least 138.6 kbp, however, the upper size limit of conjugal transfer to eukaryotes has still yet to be determined. The protocol may be adjusted for transfer to other prokaryotic or eukaryotic organisms as well. For example, some culturing methods for microanaerobic and anaerobic bacteria rely on coating or mixing cells with a semi-solid agar media layer for growth and certain algae have been cultured on plates when allowed to grow within the agar layer of the plate itself [29,30,31]. Using an adapted version of this conjugation protocol may allow engineering of species that require semi-solid or solid media to grow within. Moreover, protocols for a more accurate enumeration of colony-forming units rely on submerging cells in agar and allowing an increased number of smaller colonies to grow and be counted per plate [32]. By eliminating the need for plating with a spreader, a more accurate count of cells in a sample can be made by removing the error arising from cells sticking to the spreader. Thus, with this optimized conjugation protocol, a more accurate enumeration of transconjugants can be estimated for experimental conditions. The result is a simple, accurate protocol for conjugal transfer to *S. cerevisiae* that permits the transfer of large plasmids of at least up to 138.6 kbp.

## 4. Materials and Methods

### 4.1. Strains and Growth Conditions

NEB 5-alpha Electrocompetent *Escherichia coli* (New England Biolabs Ltd., #C2987) and Transformax Epi300 Electrocompetent *E. coli* (Lucigen, #EC300110, Madison, WI, USA) were grown in Luria-Bertani (LB) media supplemented with the appropriate antibiotic(s), chloramphenicol (30 µg/mL) and/or gentamicin (40 µg/mL). *Saccharomyces cerevisiae* VL6–48 (ATCC no. MYA-3666) was grown in 2x yeast extract/peptone/dextrose media (YPD) supplemented with 200 µg/mL adenine hemisulfate (Sigma-Aldrich, #A2545, St. Louis, MO, USA) (named YPDA) and 100 µg/mL ampicillin. Transformed spheroplasts were grown in complete minimal (CM) glucose media lacking histidine and uracil (Teknova, #C7221, Hollister, CA, USA) supplemented with adenine hemisulfate (100 µg/mL) and 1 M D-sorbitol. For yeast conjugation experiments, either CM glucose media lacking histidine supplemented with 60 µg/mL adenine (Teknova, #C7112, Hollister, CA, USA) or CM glucose media lacking histidine and uracil supplemented with adenine hemisulfate (100 µg/mL) was used when appropriate.

### 4.2. Construction of pTA-Mob 2.0 Plasmid

The first iteration of the pTA-Mob 2.0 plasmid was generated by PCR amplification of pTA-Mob [35] in nine overlapping fragments (primers, D501F/R–D509F/R); amplification of *HIS3-CEN6-ARSH4* (D510F/R) and *URA3* (D512F/R) from a Designer Microbes Inc. plasmid (pDMI-1.0, unpublished); and the RK2/RP4 origin of transfer sequence (oriT) (D511F/R) from p0521s [10] (see Supplementary Figure S1). In the final optimized protocol, the pTA-Mob 2.0 plasmid was generated by amplifying the plasmid as ten overlapping fragments using primers fragment_1F/R–fragment_10F/R. Primer sequences are listed in Supplementary Table S1.

Each fragment was individually amplified in a 50 µL PCR reaction using PrimeSTAR GXL polymerase (Takara Bio Inc., #R050A, Kusatsu, Shiga-ken, Japan), 1 µL of template DNA (see specific concentrations below), and the respective forward and reverse primers at a final concentration of 0.2 µM. For the final optimized protocol, 1 µL of 10 ng/µL template plasmid was used for fragments 1–6 and 8–10, while 1 µL of 50 ng/µL template plasmid was used for fragment 7 due to poor initial amplification. (Note, for the initial 12 fragment assembly each fragment was amplified using 1–2 ng/µL of template DNA.) The PCR programming for the optimized protocol was as follows: 25 cycles of 98 °C for 10 s, 61 °C for 15 s, and 68 °C for 70 s, followed by 1 cycle of 68 °C for 60 s, ending with an infinite hold at 12 °C. Amplification was confirmed using agarose gel electrophoresis by running 1 µL of PCR product on a 1.4% agarose (*w*/*v*) gel.

To eliminate the template DNA from the PCR products, each reaction was treated with 10 units (0.5 µL) of DpnI restriction endonuclease (New England Biolabs Ltd., #R0176), incubated at 37 °C for 30 min, and deactivated for 20 min at 80 °C. Fragments were then purified using the EZ-10 Spin Column PCR Products Purification Kit (BioBasic Inc., #BS363, Markham, ON, Canada) and diluted to approximately 60 ng/µL in nuclease-free water. Equimolar quantities of each of the ten purified fragments were mixed into a single 1.5 mL microcentrifuge tube (with a total volume of ~20 µL). For negative controls, 20 µL of nuclease-free water was used. As additional negative controls, partial assembly mixes containing either fragment 1, fragment 2, or fragment 3 were used.

The purified fragments were assembled using yeast spheroplast transformation as described in Karas et al. [47] with the exception that the bacterial culture was replaced by mixtures of DNA fragments. Following the polyethylene glycol treatment and recovery, 100 µL of transformed spheroplasts were added to 8 mL of molten CM glucose media lacking histidine and uracil supplemented with adenine hemisulfate and with 1 M D-sorbitol and 2% agar. After mixing the cells by inversion, the media was poured directly into a Petri dish. Plates were incubated at 30 °C for 24 h prior to the addition of 8 mL of liquid CM glucose media lacking histidine and uracil supplemented with adenine hemisulfate. After an additional incubation for three days at 30 °C, the liquid layer was transferred to a 15 mL centrifuge tube. Plasmid isolation was then carried out according to Karas et al. [10] and pelleted DNA was resuspended in 50 µL of elution buffer (Qiagen, #19086, Hilden, NRW, Germany).

For the initial 12-fragment assembly, 1 µL of isolated pooled yeast DNA was added to 30 µL of NEB 5-alpha electrocompetent *E. coli* cells in a 1.5 mL microcentrifuge tube on ice. The mixture was transferred to a cold 1 mm electroporation cuvette and electroporated at 1.8 kV using the BioRad GenePulser. For the final optimized protocol, 25 µL of TransforMax Epi300 Electrocompetent *E. coli* cells were mixed on ice with 1 µL DNA isolated from pooled yeast transformants. The mixture was transferred to a cold 2 mm electroporation cuvette and electroporated at 2.5 kV. Cells were recovered in 1 mL of Super Optimal broth with Catabolite repression [48] at 37 °C, shaking at 225 rpm for 1 h. Following recovery, 100 µL of the transformed cell mixture was plated on 1.5% agar (*w*/*v*) LB media plates containing gentamicin (40 µg/mL) and incubated at 37 °C overnight.

To screen for correctly assembled plasmids, individual *E. coli* colonies were tested as follows:

For the initial 12-fragment assembly, 50 *E. coli* colonies were streaked onto a fresh LB agar plate containing gentamicin (40 µg/mL) and incubated at 37 °C overnight. A second LB agar plate supplemented with chloramphenicol (30 µg/mL) was streaked with Epi300 *E. coli* containing the pAGE2.0 [9] plasmid and incubated at 37 °C overnight. The next morning, each *E. coli* colony was streaked on top of *E. coli* pAGE2.0 on nonselective LB agar plates and incubated for 3 h at 37 °C. Cells were scraped and resuspended in 200 µL liquid LB media before performing 10-fold serial dilutions from 10^−1^ to 10^−4^. Then, 5 µL of the serial dilutions for each sample were spot plated on LB agar plates containing gentamicin (40 µg/mL) and chloramphenicol (30 µg/mL) and incubated at 37 °C overnight. The ability of each *E. coli* colony to conjugate was assessed by transconjugant colony growth at each dilution the following day. One colony was selected, and the plasmid isolated from this strain was named pTA-Mob 2.0. Plasmid DNA was isolated using the EZ-10 Spin Column Plasmid DNA Miniprep Kit (BioBasic Inc., #BS413, Markham, ON, Canada) and sent for complete plasmid sequencing at the Massachusetts General Hospital DNA Core.

For the final optimized plasmid assembly protocol, 30 colonies were tested. First, the colonies were streaked on LB plates supplemented with gentamicin (40 µg/mL) and incubated at 37 °C overnight. A second LB agar plate containing chloramphenicol (30 µg/mL) was streaked with Epi300 *E. coli* containing the pAGE1.0 plasmid and incubated at 37 °C overnight [9]. The next day, overnight cultures of 3 mL LB supplemented with gentamicin (40 µg/mL) were inoculated with one of the 30 newly assembled donor *E. coli* pTA-Mob 2.0 colonies. In addition, two overnight cultures of recipient *E. coli* pAGE1.0 were grown in 3 mL LB containing chloramphenicol (30 µg/mL). As a positive control for conjugation, a 3 mL LB with gentamicin (40 µg/mL) culture of the original *E. coli* pTA-Mob 2.0 was also inoculated. Cultures were incubated overnight at 37 °C in a tube rotator. After 16 h, 100 µL of donor and 100 µL of recipient cultures were mixed, spread on nonselective LB agar plates, and incubated for 1 h at 37 °C. The following controls were also performed: (i) parental donor *E. coli* pTA-Mob 2.0 with recipient *E. coli* pAGE1.0 (positive control), (ii) parental donor *E. coli* pTA-Mob 2.0 with water (negative control), and iii) recipient *E. coli* pAGE1.0 with water (negative control). Conjugation plates were then scraped with 1 mL LB media, and cells were transferred to a 1.5 mL microcentrifuge tube. Each mixture was serially diluted 10-fold from 10^−1^ to 10^−5^ using LB media, and a 5 µL volume of each of the serial dilutions was spot plated onto LB agar plates supplemented with gentamicin (40 µg/mL) and chloramphenicol (30 µg/mL). Plates were incubated overnight at 37 °C and photographed the next day.

### 4.3. Conjugation within Solid Media

#### 4.3.1. Preparation of *E. coli*

Cultures of 50 mL of LB media supplemented with the appropriate antibiotic (40 µg/mL gentamicin for *E. coli* pTA-Mob 2.0 or 40 µg/mL gentamicin and 30 µg/mL chloramphenicol for *E. coli* pAGE1.0 [9] with pTA-Mob [35] and *E. coli* pBK-RBYV-25-2 [10,38,49] with pTA-Mob) were inoculated with freshly grown *E. coli* and incubated overnight at 37 °C and 225 rpm in 250 mL Erlenmeyer flasks. The following morning, saturated bacterial cultures were diluted 100x to a total volume of 50 mL in LB media with the appropriate antibiotic in a new flask. The diluted cultures were grown to an optical density at 600 nm (OD_600_) of 0.5, 1.0, or 2.0. The cultures were then transferred to 50 mL centrifuge tubes and pelleted for 10 min at 5000 relative centrifugal force (RCF). After centrifugation, the supernatants were decanted, and the pellets were resuspended in 1 mL LB media.

#### 4.3.2. Preparation of *S. cerevisiae*

A 50 mL overnight culture was inoculated with fresh yeast and grown at 30 °C at 225 rpm in 2x YPDA media supplemented with ampicillin (100 µg/mL) in a 250 mL Erlenmeyer flask. The culture was grown to an OD_600_ of 1.0. The culture was then transferred to a 50 mL centrifuge tube and pelleted for 10 min at 5000 RCF. After decanting the supernatant, the cell pellet was resuspended in 1 mL sterile double deionized H_2_O (sddH_2_O).

#### 4.3.3. Conjugation within Solid Media Procedure

Mixtures of 25, 50, or 100 µL of *E. coli* cell suspension with 50, 100, or 200 µL of *S. cerevisiae* cell suspension were brought to a final volume of 1 mL with sddH_2_O in a 1.5 mL microcentrifuge tube. Centrifuge tubes containing 5 mL of either molten CM glucose media lacking histidine and uracil with 1%, 1.5%, or 2% agar (*w*/*v*) or molten CM glucose media lacking histidine supplemented with adenine with 2% agar were prepared and held in a water bath set to either 55 °C, 60 °C, or 65 °C. The tubes were removed from the water bath, then, the cell mixture was added to the molten media and inverted three times to mix. Cells resuspended in agar were then poured onto the respective base plate of 25 mL CM glucose media lacking histidine and uracil with 2% agar or CM glucose media lacking histidine with 2% agar preincubated at 37 °C. After a 20 min drying period, conjugation plates were incubated at 30 °C for four days. Successful transconjugant *S. cerevisiae* colonies were then counted under 5x magnification using a Zeiss Discovery.V8 SteREO microscope.

To quantify conjugation frequency, *S. cerevisiae* culture was serially diluted and plated onto YPDA 1% agar plates. Colonies were counted after two days of incubation at 30 °C.

#### 4.3.4. Confirmation of Successfully Transferred Plasmids

Single colonies were picked from the agar and streaked onto CM glucose media lacking histidine with 2% agar plates containing ampicillin (100 µg/mL). A total of three successive passages were performed, streaking a small portion of cells after a two-day incubation at 30 °C each time. Following the second passage, a small portion of cells was streaked onto LB plates containing gentamicin (40 µg/mL) to test for surviving donor *E. coli*. After the third passage, a small portion of each streak was resuspended in 100 µL TE buffer (pH 8) and incubated at 98 °C for 15 min. Multiplex PCR was performed using a Qiagen Multiplex PCR Kit (Qiagen, #206143) and the heat-treated suspension as template for primers (Supplementary Table S1) that bind to pAGE1.0, pTA-Mob 2.0, and pBK-RBYV-25-2 in locations distributed around each plasmid. The multiplex PCR conditions were as follows: a 95 °C hot start for 15 min, followed by 35 cycles of 94 °C for 30 s, 60 °C for 90 s, and 72 °C for 45 s, followed by a final extension at 72 °C for 10 min, and a 12 °C infinite hold. Multiplex amplification was confirmed using agarose gel electrophoresis with 2 µL of each multiplex PCR reaction run on a 2% agarose (*w*/*v*) gel.

## 5. Conclusions

We developed protocol for *cis* and *trans* conjugal transfer from *E. coli* to *S. cerevisiae* within solid media. Using this protocol, we transferred plasmids up to 138.6 kbp. Additionally, we developed PCR-based synthesis pipeline to generate custom conjugative plasmids such as the plasmid pTA-Mob 2.0. These tools improve how we create and deliver DNA by conjugation from prokaryotic to eukaryotic cells.

## Supplementary Materials

The following are available online at https://www.mdpi.com/1422-0067/20/20/5212/s1.

## References

[1] Cabezón E., Ripoll-Rozada J., Peña A., De La Cruz F., Arechaga I. (2014). Towards an integrated model of bacterial conjugation. FEMS Microbiol. Rev..

[2] Chilton M.-D., Drummond M.H., Merlo D.J., Sciaky D., Montoya A.L., Gordon M.P., Nester E.W. (1977). Stable incorporation of plasmid DNA into higher plant cells: The molecular basis of crown gall tumorigenesis. Cell.

[3] Christie P.J. (1997). Agrobacterium tumefaciens T-complex transport apparatus: A paradigm for a new family of multifunctional transporters in eubacteria. J. Bacteriol..

[4] Stachel S.E., Zambryski P.C. (1989). Generic trans-kingdom sex?. Nature.

[5] Heinemann J.A., Sprague G.F. (1989). Bacterial conjugative plasmids mobilize DNA transfer between bacteria and yeast. Nature.

[6] Bundock P., den Dulk-Ras A., Beijersbergen A., Hooykaas P.J. (1995). Trans-kingdom T-DNA transfer from Agrobacterium tumefaciens to *Saccharomyces cerevisiae*. EMBO J..

[7] Bates S., Cashmore A.M., Wilkins B.M. (1998). IncP plasmids are unusually effective in mediating conjugation of Escherichia coli and *Saccharomyces cerevisiae*: Involvement of the tra2 mating system. J. Bacteriol..

[8] Moriguchi K., Yamamoto S., Ohmine Y., Suzuki K. (2016). A Fast and Practical Yeast Transformation Method Mediated by Escherichia coli Based on a Trans-Kingdom Conjugal Transfer System: Just Mix Two Cultures and Wait One Hour. PLoS ONE.

[9] Brumwell S.L., MacLeod M.R., Huang T., Cochrane R.R., Meaney R.S., Zamani M., Matysiakiewicz O., Dan K.N., Janakirama P., Edgell D.R. (2019). Designer Sinorhizobium meliloti strains and multi-functional vectors enable direct inter-kingdom DNA transfer. PLoS ONE.

[10] Karas B.J., Diner R.E., Lefebvre S.C., McQuaid J., Phillips A.P.R., Noddings C.M., Brunson J.K., Valas R.E., Deerinck T.J., Jablanovic J. (2015). Designer diatom episomes delivered by bacterial conjugation. Nat. Commun..

[11] Diner R.E., Bielinski V.A., Dupont C.L., Allen A.E., Weyman P.D. (2016). Refinement of the Diatom Episome Maintenance Sequence and Improvement of Conjugation-Based DNA Delivery Methods. Front. Bioeng. Biotechnol..

[12] Slattery S.S., Diamond A., Wang H., Therrien J.A., Lant J.T., Jazey T., Lee K., Klassen Z., Desgagné-Penix I., Karas B.J. (2018). An Expanded Plasmid-Based Genetic Toolbox Enables Cas9 Genome Editing and Stable Maintenance of Synthetic Pathways in *Phaeodactylum tricornutum*. ACS Synth. Biol..

[13] Wang H., Slattery S., Karas B., Edgell D. (2018). Delivery of the Cas9 or TevCas9 System into *Phaeodactylum tricornutum* via Conjugation of Plasmids from a Bacterial Donor. Bio-Protocol.

[14] Waters V.L. (2001). Conjugation between bacterial and mammalian cells. Nat. Genet..

[15] Kunik T., Tzfira T., Kapulnik Y., Gafni Y., Dingwall C., Citovsky V. (2001). Genetic transformation of HeLa cells by Agrobacterium. Proc. Natl. Acad. Sci. USA.

[16] Fernández-González E., de Paz H.D., Alperi A., Agúndez L., Faustmann M., Sangari F.J., Dehio C., Llosa M. (2011). Transfer of R388 derivatives by a pathogenesis-associated type IV secretion system into both bacteria and human cells. J. Bacteriol..

[17] Schröder G., Schuelein R., Quebatte M., Dehio C. (2011). Conjugative DNA transfer into human cells by the VirB/VirD4 type IV secretion system of the bacterial pathogen Bartonella henselae. Proc. Natl. Acad. Sci. USA.

[18] Aune T.E.V., Aachmann F.L. (2010). Methodologies to increase the transformation efficiencies and the range of bacteria that can be transformed. Appl. Microbiol. Biotechnol..

[19] Heinze S., Kornberger P., Grätz C., Schwarz W.H., Zverlov V.V., Liebl W. (2018). Transmating: Conjugative transfer of a new broad host range expression vector to various Bacillus species using a single protocol. BMC Microbiol..

[20] Karas B.J., Jablanovic J., Sun L., Ma L., Goldgof G.M., Stam J., Ramon A., Manary M.J., Winzeler E.A., Venter J.C. (2013). Direct transfer of whole genomes from bacteria to yeast. Nat. Methods.

[21] Hill K.E., Top E.M. (1998). Gene transfer in soil systems using microcosms. FEMS Microbiol. Ecol..

[22] Brophy J.A.N., Triassi A.J., Adams B.L., Renberg R.L., Stratis-Cullum D.N., Grossman A.D., Voigt C.A. (2018). Engineered integrative and conjugative elements for efficient and inducible DNA transfer to undomesticated bacteria. Nat. Microbiol..

[23] Ronda C., Chen S.P., Cabral V., Yaung S.J., Wang H.H. (2019). Metagenomic engineering of the mammalian gut microbiome in situ. Nat. Methods.

[24] Hamilton T.A., Pellegrino G.M., Therrien J.A., Ham D.T., Bartlett P.C., Karas B.J., Gloor G.B., Edgell D.R. (2019). Efficient inter-species conjugative transfer of a CRISPR nuclease for targeted bacterial killing. Nat. Commun..

[25] Itaya M., Sato M., Hasegawa M., Kono N., Tomita M., Kaneko S. (2018). Far rapid synthesis of giant DNA in the Bacillus subtilis genome by a conjugation transfer system. Sci. Rep..

[26] Zechner E.L., Moncalián G., de la Cruz F., Backert S., Grohmann E. (2018). Relaxases and Plasmid Transfer in Gram-Negative Bacteria. Type IV Secretion in Gram-Negative and Gram-Positive Bacteria.

[27] Christie P.J. (2001). Type IV secretion: Intercellular transfer of macromolecules by systems ancestrally related to conjugation machines. Mol. Microbiol..

[28] Bradley D.E., Taylor D.E., Cohen D.R. (1980). Specification of surface mating systems among conjugative drug resistance plasmids in Escherichia coli K-12. J. Bacteriol..

[29] Mason J.H. (1953). Isolation of anaerobic bacteria by a modified shake method. J. Gen. Microbiol..

[30] Nagasaki K., Imai I. (1994). Solid-phase culture of marine phytoflagellates. Bull. Jpn. Soc. Mircobial Ecol..

[31] Lakeman M.B., Cattolico A.R.A. (2007). Cryptic Diversity in Phytoplankton Cultures is Revealed Using a Simple Plating Technique. J. Phycol..

[32] Koch A.L., Gerhard P., Murray R.G.E., Wood W.A., Krieg N.R. (2007). Growth Measurement. Methods for General and Molecular Biology.

[33] Zhu Y.O., Sherlock G., Petrov D.A. (2016). Whole Genome Analysis of 132 Clinical *Saccharomyces cerevisiae* Strains Reveals Extensive Ploidy Variation. G3 Genes Genomes Genet..

[34] Gajdács M., Ábrók M., Lázár A., Burián K. (2019). Comparative Epidemiology and Resistance Trends of Common Urinary Pathogens in a Tertiary-Care Hospital: A 10-Year Surveillance Study. Medicina.

[35] Strand T.A., Lale R., Degnes K.F., Lando M., Valla S. (2014). A new and improved host-independent plasmid system for RK2-based conjugal transfer. PLoS ONE.

[36] Pansegrau W., Lanka E., Barth P.T., Figurski D.H., Guiney D.G., Haas D., Helinski D.R., Schwab H., Stanisich V.A., Thomas C.M. (1994). Complete Nucleotide Sequence of Birmingham IncPα Plasmids: Compilation and Comparative Analysis. J. Mol. Biol..

[37] Karas B.J., Moreau N.G., Deerinck T.J., Gibson D.G., Venter J.C., Smith H.O., Glass J.I. (2019). Direct Transfer of a Mycoplasma mycoides Genome to Yeast Is Enhanced by Removal of the Mycoides Glycerol Uptake Factor Gene glpF. ACS Synth. Biol..

[38] Karas B.J., Molparia B., Jablanovic J., Hermann W.J., Lin Y.-C., Dupont C.L., Tagwerker C., Yonemoto I.T., Noskov V.N., Chuang R.-Y. (2013). Assembly of eukaryotic algal chromosomes in yeast. J. Biol. Eng..

[39] Miyano M., Tanaka K., Ishikawa S., Takenaka S., Miguel-Arribas A., Meijer W.J.J., Yoshida K.I. (2018). Rapid conjugative mobilization of a 100kb segment of Bacillus subtilis chromosomal DNA is mediated by a helper plasmid with no ability for self-transfer. Microb. Cell Fact..

[40] Moriguchi K., Yamamoto S., Tanaka K., Kurata N., Suzuki K. (2013). Trans-Kingdom Horizontal DNA Transfer from Bacteria to Yeast Is Highly Plastic Due to Natural Polymorphisms in Auxiliary Nonessential Recipient Genes. PLoS ONE.

[41] Quandt J., Clark R.G., Venter A.P., Clark S.R., Twelker S., Hynes M.F. (2004). Modified RP4 and Tn5-Mob derivatives for facilitated manipulation of large plasmids in Gram-negative bacteria. Plasmid.

[42] Potapov V., Ong J.L. (2017). Examining Sources of Error in PCR by Single-Molecule Sequencing. PLoS ONE.

[43] Zhang S., Chen T., Jia J., Guo L., Zhang H., Li C., Qiao R. (2019). Establishment of a highly efficient conjugation protocol for Streptomyces kanamyceticus ATCC12853. MicrobiologyOpen.

[44] Zeng X., Ardeshna D., Lin J. (2015). Heat Shock-Enhanced Conjugation Efficiency in Standard Campylobacter jejuni Strains. Appl. Environ. Microbiol..

[45] Kirk J.A., Fagan R.P. (2016). Heat shock increases conjugation efficiency in Clostridium difficile. Anaerobe.

[46] Heinemann J.A., Sprague G.F. (1991). Transmission of Plasmid DNA to Yeast by Conjugation with Bacteria. Methods Enzymol..

[47] Karas B.J., Jablanovic J., Irvine E., Sun L., Ma L., Weyman P.D., Gibson D.G., Glass J.I., Venter J.C., Hutchison C.A. (2014). Transferring whole genomes from bacteria to yeast spheroplasts using entire bacterial cells to reduce DNA shearing. Nat. Protoc..

[48] Hanahan D. (1983). Studies on transformation of Escherichia coli with plasmids. J. Mol. Biol..

[49] Diner R.E., Noddings C.M., Lian N.C., Kang A.K., McQuaid J.B., Jablanovic J., Espinoza J.L., Nguyen N.A., Anzelmatti M.A., Jansson J. (2017). Diatom centromeres suggest a mechanism for nuclear DNA acquisition. Proc. Natl. Acad. Sci. USA.

