# Supplementary material for: Trans-Kingdom Conjugation within Solid Media from Escherichia coli to Saccharomyces cerevisiae
Source: Int J Mol Sci. 2019 Oct 21;20(20):5212. doi: 10.3390/ijms20205212 (PMC6829330; doi:10.3390/ijms20205212)
Supplement: Supplementary file 1 [file ijms-20-05212-s001.pdf]

International Journal of
Molecular Sciences

MDPI

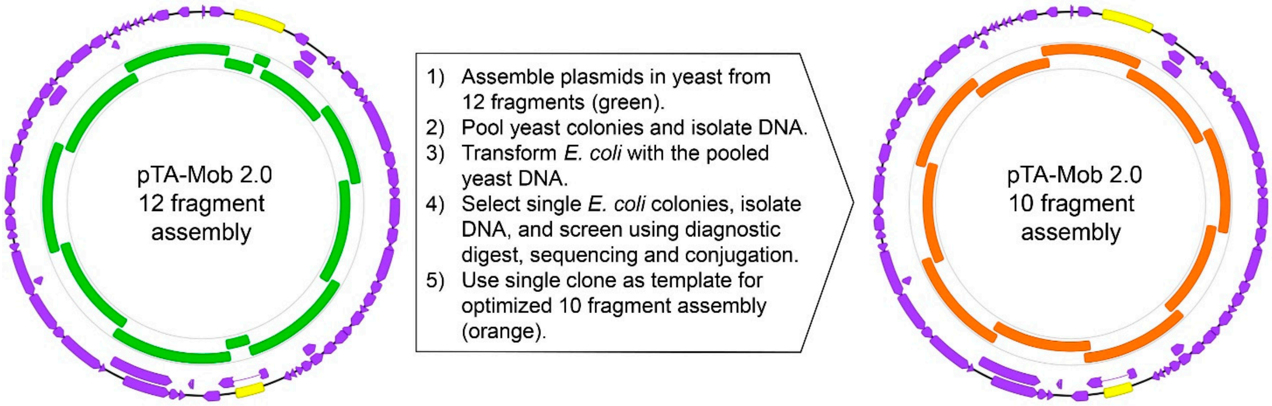

Supplementary Figure S1. Overview of the original (12 fragment) and final (10 fragment) pTA-Mob 2.0 assembly. The plasmid maps were generated using Geneious version 2019.2, created by Biomatters.

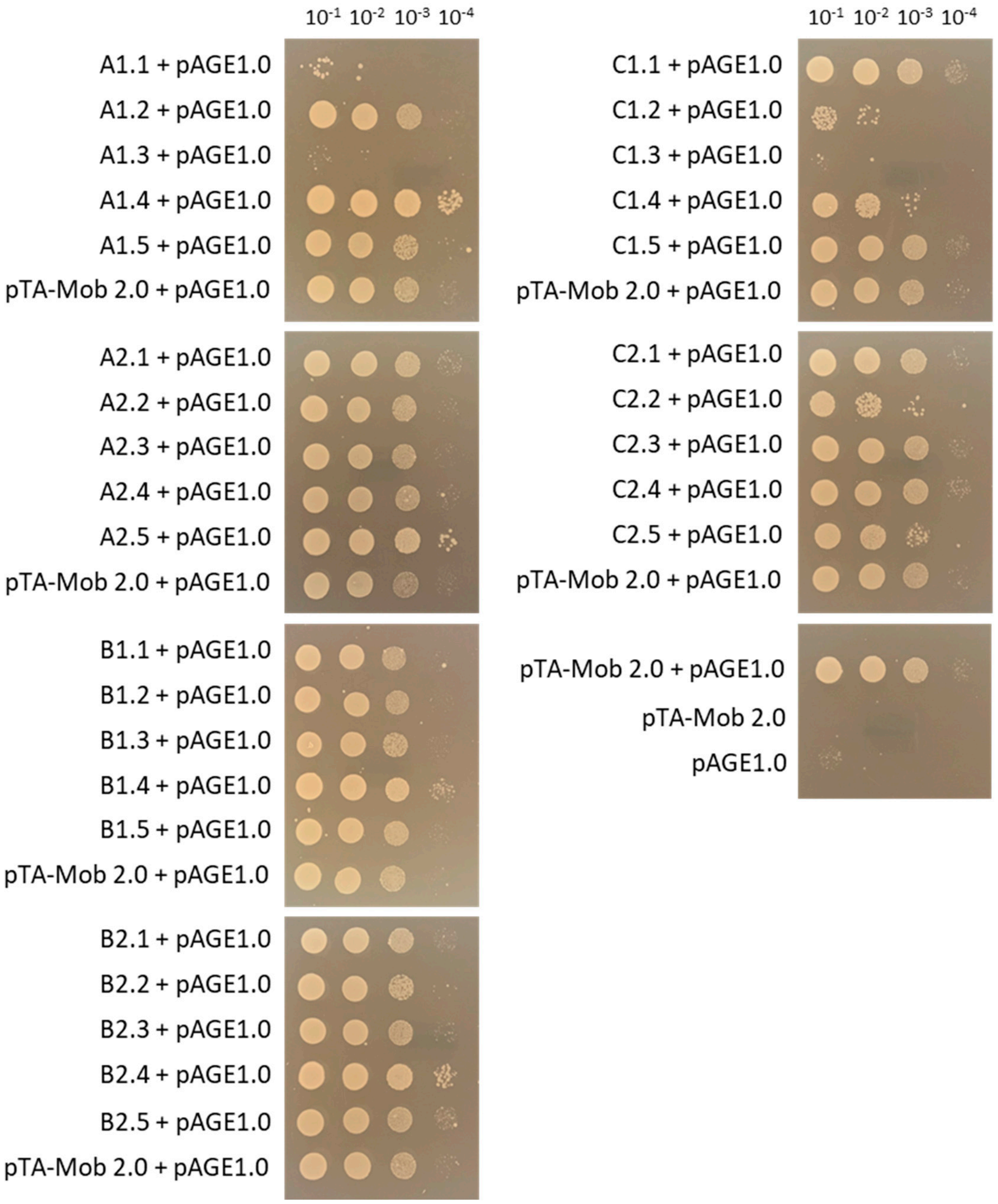

Supplementary Figure S2. Conjugation results for the 30 assembled donor *E. coli* pTA-Mob 2.0 clones to recipient *E. coli* pAGE1.0. The *E. coli* strain containing the parental pTA-Mob 2.0 plasmid was used as a template for amplification of the new pTA-Mob 2.0 clones and included as a positive control for conjugation. For negative controls, only the parental donor pTA-Mob 2.0 plasmid or the recipient pAGE1.0 were used.

Supplementary Note S1. Optimized protocol for conjugation to *S. cerevisiae* within solid media

Preparation of Epi300 *E. coli* pTA-Mob 2.0:

Inoculate a 50 mL culture in LB media supplemented with 40 μg/mL gentamicin with fresh *E. coli* pTA-Mob 2.0. The overnight culture should be grown at 225 rpm in a 250 mL Erlenmeyer flask at 37°C. The next morning, dilute the culture 100x by transferring 500 μL of overnight culture into a new flask of 50 mL LB with 40 μg/mL gentamicin. Continue growing the culture until it reaches an OD_{600} of approximately 1.0. It is better to harvest the donor *E. coli* at an OD_{600} lower than or at 1.0 if possible, as opposed to over 1.0. Note: at these specified conditions and strains, it takes approximately 3 hours for the 100x diluted *E. coli* culture to

reach the target OD_{600}. Transfer the culture to a 50 mL centrifuge tube and pellet cells for 10 minutes at 5,000 RCF at 10°C. If this speed cannot be met, spinning at 3,000 RCF for 15 minutes or longer will suffice. Resuspend the cell pellet in 1 mL LB media.

Preparation of *S. cerevisiae* VL6-48:

Inoculate a 50 mL culture in 2x YPDA media supplemented with 100 µg/mL ampicillin with fresh *S. cerevisiae* VL6-48. Time the culture to reach an OD_{600} of approximately 1.0 for when the donor *E. coli* culture will also hit the target OD_{600} (as described above). Transfer the culture to a 50 mL centrifuge tube and pellet cells for 10 minutes at 5,000 RCF at 10°C. As described before, if this speed cannot be met, spinning at 3,000 rcf for 15 minutes or longer will suffice. Resuspend the cell pellet in sterile ddH_2O.

Preparation of molten media and base plates:

Choose the complete minimal (CM) glucose media lacking the supplement you are selecting with for recipient *S. cerevisiae*. In this protocol with pTA-Mob 2.0, melt CM glucose media lacking histidine and uracil supplemented with adenine hemisulfate with 2% agar (w/v). Aliquot 5 mL of molten selective media into 15 mL centrifuge tubes. Prepare extra tubes for control samples. Transfer molten media aliquots to a water bath set to 60°C for holding until conjugation samples are prepped and ready. Additionally, make 25 mL CM glucose media lacking histidine and uracil supplemented with adenine hemisulfate with 2% agar (w/v) plates. Once dry, transfer the base plates to an incubator set to 37°C or higher for warming at least 1 hour prior to conjugation. Note: during the waiting period for the diluted donor *E. coli* culture to reach the target OD_{600} is usually when these media are best prepared. Plates can be made in advance, but molten media for the top layer should not be made and held more than 12 hours in advance to avoid solidification or contamination.

Conjugation within solid media procedure:

Mix 100 µL of donor *E. coli* pTA-Mob 2.0 resuspension with 100 µL of recipient *S. cerevisiae* resuspension in a microcentrifuge tube. Bring the mixture up to a volume of 1 mL with sterile ddH_2O. (Note: if larger and fewer colonies are desired, use a smaller percentage of the 100 µL/ 100 µL ratio such as 60 µL/60 µL. Ensure the end mixture is a total volume of 1 mL and contains 10% LB.) Once all samples have been prepared, only work with a single molten media aliquot and plate at a time to avoid cooling. If a distance needs to be traversed from the water bath to the bench or biosafety hood, take some water out of the 60°C water bath in a beaker and use it to keep the molten media aliquot at the appropriate temperature while moving. Take the base plate out of the holding incubator as well. Transfer the entire 1 mL cell mixture from the microcentrifuge tube into the 5 mL molten selective agar media in the centrifuge tube and invert three times to mix. Pour the mixture onto the base plate. Rotate the base plate while pouring to ensure an even distribution of the new cell-agar layer. Let the new layer dry for at least 20 minutes, then move conjugation plates to 30°C for incubation. Colonies will begin to appear after 48 hours. If incubating plates for longer than 72 hours, either parafilm the plates or keep the plates inside of a sealed bag to prevent the plates from drying out. After incubation, colonies can be picked and streaked onto a new plate using a micropipette tip.

Supplementary Table S1. Primers Used in This Study

Primer	Sequence	Product Size (bp)
**Primers for Initial Assembly of pTA-Mob 2.0**		
**D501F**	GTCGGCAAAGCAAACATCCATCGAACAGCCTTGCGTGTGGGGGTCCACGCCTTCGACCAG	**6138**
**D501R**	GCCGTGTTCAAACGATACCTGGCAGTGACTCCTAGCGCTCACCAAGCTCTTATTAATTAAGTTAAACGCCTGGTGCTACG	

D502F	GGTCAACCAGCCCTTGAAAC	6413
D502R	ACCGCACTCACCTATTTCGT	
D503F	GATCCAGCCGACCAGGCTTT	6400
D503R	GGCAGCTCTTGCGATTTCAG	
D504F	TTGTGCAGCTCGGAGACTTT	6440
D504R	GCGATTGAGGACCGCATCT	
D505F	GGCAGCGTTGGGTCCTGGCCACGGGTGCGCATGATCGTGCTCCTGTCGTTGAGGCGCGCCAACTTTGCGGTTAATACGCT	7006
D505R	GCGCCGAGGTCGCCAACATCAAAGACAACGGCCTCAACATGAAGATCGACACGCACCCTT	
D506F	CTATCCCTATGACCTGGCGGCCTGGCGGTTGCGCGGGCACATTCGCAACGACAGGGGCGA	6549
D506R	CGCCGCGTGCGGCTGCTGGAGATGGCGGACGCGATGGATATGTTCTGCCAAGGGCGCGCCAACTTTGCACCGTTGCCCGG	
D507F	TCGGTGCGCTTCCTGTTC	6459
D507R	GGTGATCCGGCCTTGCTTC	
D508F	CCCGAAGCCCTTGATCTGTT	4814
D508R	CAGGCGCATGTGGTAGCTGC	
D509F	AGAAGAGGCACTTCGAGCTGTAAGTACATCACCGACGAGCAAGGCAAGACGATCGAGCTCTAGGAGTGCGGTTGGAACGT	4541
D509R	AAATACTCCTTACAGGGTTTTGGTGATGTACTGGCCGTTCTCGTAACCAAGAACTTCGAG	
D510F	GCCATTCATCCGCTTATTATCACTTATTCAGGCGTAGCACCAGGCGTTTAACTTAATTAATAAGAGCTTGGTGAGCGCTA	1760
D510R	TATACCGAAAAAATCGCTATAATGACCCCGAAGCAGGGTTATGCAGCGGAAGATACTAGTGGATCGCTTGCCTGTAACTT	
D511F	ATTATTCCATCATTAAAAGATACGAGGCGCGTGTAAGTTACAGGCAAGCGATCCACTAGTATCTTCCGCTGCATAACCCT	891
D511R	TCCTGCTCGTGATCGGGAGTATCTGGCTGGGCCAACGTTCCAACCGCACTCCTAGAGCTCGATCGTCTTGCCTTGCTCGT	
D512F	CAGCACGACTGGGGCGAAGTTGAGGTGGAGGTTGCCGGGCAACGGTGCAAAGTTGGCGCGCCCTTGGCAGAACATATCCA	1404
D512R	TGCGGCGAAGACATGGAAGCGGCGGGAGAACCCCAGCGTATTAACCGCAAAGTTGGCGCGCCTCAACGACAGGAGCACGA	
**Primers to Assemble Optimized pTA-Mob 2.0**		
Fragment_1_F	TGCCGCCGCGCGCATGGTCGTAATGGGACCGATAGCCCGT	5782
Fragment_1_R	TTTAACCTACTTCCTTTGGTTCCGGGGGATCTCGCGACTC	
Fragment_2_F	ATCGAAGAGAAGCAGGACGA	6373
Fragment_2_R	TGCTGGTCCATGAAGATGAA	
Fragment_3_F	TCGAGCTGATGTTTGACGAC	6137
Fragment_3_R	GGACTTGAGGTTGCTCTGCT	
Fragment_4_F	GGACCAGGCGCAGTCCACCATCAACGGCCTGATGAGCGCC	6000

**Fragment_4_R**	ATCGGCGTGAAGCCCAACAGGGCCA	
**Fragment_5_F**	GTGGACATTGGTTTCAGCAA	**6273**
**Fragment_5_R**	AGCTCATGCATCACAACAGC	
**Fragment_6_F**	GAGCAATGGATAGCCGATGT	**6206**
**Fragment_6_R**	AAGCGATGAATGATCCCAAG	
**Fragment_7_F**	TGTAACGCTTCCCGGTAGTC	**6295**
**Fragment_7_R**	CATTGCAAAGCGACTGATGT	
**Fragment_8_F**	GATCCGCTCCTTGAACTCTG	**6259**
**Fragment_8_R**	AGGCCCTTGCCAATGAAT	
**Fragment_9_F**	TTCTTTGAATGCGCGGGCGTCCTGGTGAGCGTAGTCCAGC	**6000**
**Fragment_9_R**	CGTTCCCGCCTGCCCCTGATTGGCCCGCTGATCGACCGCT	
**Fragment_10_F**	AATGTTGCAAGGCGATCAG	**5745**
**Fragment_10_R**	AGCCCTCCCGTATCGTAGTT	
**MPX Primers for pAGE1.0**		
**pAGE_MPX_191_F**	TTGGCCCTCACTGACAGATGAG	**191**
**pAGE_MPX_191_R**	CTTATCCCCAGGCTTGTCCACA	
**pAGE_MPX_288_F**	GGATGCGTGTCCTTCAGTGA	**288**
**pAGE_MPX_288_R**	ACAACTGCCGCAGTACTCAA	
**pAGE_MPX_394_F**	TGAACAGGCCATTGATCAACGC	**394**
**pAGE_MPX_394_R**	GATCATCCAGTGCGTCCTCAGT	
**MPX Primers for pTA-Mob 2.0**		
**Mob_MPX_192_F**	CACCAGGACAGTAACGACCCAT	**192**
**Mob_MPX_192_R**	TGGACGAATTGAACACGCATCG	
**Mob_MPX_255_F**	ACCATGAATCGCGGCATTTTGT	**255**
**Mob_MPX_255_R**	AACCAGGAAGATCAGGGTTCGG	
**Mob_MPX_408_F**	CCGCTGCGAACACCACTACGTT	**408**
**Mob_MPX_408_R**	TGCCTCGGCAAAATCCTTGCGT	
**Mob_MPX_511_F**	CGTATAGCTCGGTGGTGTCGAT	**511**
**Mob_MPX_511_R**	ACCGACAACCTGCACATCCATA	
**MPX Primers for pBK-RBYV-25-2**		
**25_MPX_158_F**	GCCCTAGGATTACCTACGCTGG	**158**
**25_MPX_158_R**	TTTGAGGATTCGTCGTTGCTGC	
**25_MPX_195_F**	GACTTTTGGGTTCACCGCGTAG	**195**
**25_MPX_195_R**	CGCCATCATATCCGTGACGTTG	
**25_MPX_257_F**	CGGGTCGAAAACATGGTGGAAG	**257**
**25_MPX_257_R**	TTCCCAAGCGTAATTGTGCGAC	

25_MPX_299_F	ACGAACCGCAGAGAAGACTACC	299
25_MPX_299_R	GTGAAGAATTTCCCGTCGCGTT	
25_MPX_398_F	CCGGCTGGTCGCTAATCGTTGAGTGC	398
25_MPX_398_R	GGGCGAGGTGGCTTCTCTTATGGCAACCG	

Supplementary Table S2. Mutations in pTA-Mob 2.0 clones synthesized by the optimized protocol**.** Plasmid DNA was sequenced by CCIB DNA Core Facility at Massachusetts General Hospital (Cambridge, MA). Sequences were aligned using Clustal Omega and disagreements with the original pTA-Mob 2.0 sequence were identified using Geneious.

pTA-Mob 2.0 Clone	Point Mutations			Gap Mutations		Total
	Synonymous	Non-synonymous	Non-coding	Insertions	Deletions	
**A1.2**	2	1	1	0	0	**4**
**A2.3**	2	2	7	0	1	**12**
**C1.5**	1	2	0	0	0	**3**
